# COVID-19 in patients with HIV-1 infection: a single-centre experience in northern Italy

**DOI:** 10.1007/s15010-020-01492-7

**Published:** 2020-08-03

**Authors:** Leonardo Calza, Isabella Bon, Marina Tadolini, Marco Borderi, Vincenzo Colangeli, Lorenzo Badia, Gabriella Verucchi, Giada Rossini, Caterina Vocale, Paolo Gaibani, Pierluigi Viale, Luciano Attard

**Affiliations:** 1 Department of Medical and Surgical Sciences, Unit of Infectious Diseases, “Alma Mater Studiorum” University of Bologna, S. Orsola-Malpighi Hospital, via G. Massarenti 11 - I-40138, Bologna, Italy; 2 Department of Experimental, Diagnostic and Specialty Medicine, Unit of Microbiology, “Alma Mater Studiorum” University of Bologna, S. Orsola Hospital, Bologna, Italy

**Keywords:** Coronavirus, HIV, Comorbidity, Pneumonia, Protease inhibitors

## Abstract

**Background:**

Since the end of February 2020, the Coronavirus Disease 2019 (COVID-19) outbreak rapidly spread throughout Italy and other European countries, but limited information has been available about its characteristics in HIV-infected patients.

**Methods:**

We have described a case series of patients with HIV infection and COVID-19 diagnosed at the S.Orsola Hospital (Bologna, Italy) during March and April, 2020.

**Results:**

We reported a case series of 26 HIV-infected patients with COVID-19. Nineteen subjects were men, the median age was 54 years, 73% of patients had one or more comorbidities. Only 5 patients with interstitial pneumonia were hospitalized, but there were no admissions to intensive care unit and no deaths.

**Conclusions:**

In our experience, COVID-19 associated with HIV infection had a clinical presentation comparable to the general population and was frequently associated with chronic comorbidities.

## Introduction

In early December 2019, an interstitial pneumonia of unknown origin emerged in Wuhan city, the capital of Hubei province in China. The pathogen has been identified as a novel betacoronavirus, which has subsequently been named Severe Acute Respiratory Syndrome Coronavirus 2 (SARS-CoV-2), and the illness caused by SARS-CoV-2 has been defined Coronavirus disease 2019 (COVID-19) [1, 2].

During the first 2 months of the outbreak, COVID-19 spread rapidly throughout China and caused varying degrees of illness. In a retrospective analysis of 1099 patients with laboratory-confirmed COVID-19 from 30 provinces in China, the median incubation period was 4 days, the most common symptoms were fever and cough, and the most frequent laboratory abnormalities were lymphocytopenia, increased lactate dehydrogenase, and increased C-reactive protein. On chest computed tomography, ground-glass opacity was the most common radiologic finding, 5% of patients were admitted to an intensive care unit (ICU), and 1.4% died [1, 2]. In a meta-analysis of 43 studies involving 3600 patients, bilateral pneumonia was present in 73.2% of cases, the estimated proportion of severe cases was 25.6%, and the case-fatality rate was 3.6% [3].

In the following weeks, the COVID-19 outbreak became a global pandemic and on February 20, 2020, the first patient with SARS-CoV-2 pneumonia was diagnosed in Lombardy region, in northern Italy. Since then, the increasing number of cases reported throughout the country led Italy to be the second most affected country in the world, after the United States, as of the end of March 2020 [4].

A matter of debate includes frequency and seriousness of COVID-19 in immune compromised patients, such as those with human immune deficiency virus-1 (HIV-1) infection. To date, clinical data about COVID-19 in HIV-1-infected patients are lacking and descend only from a few case series [5–11].

## Case series

Between March 1, 2020 and April 30, 2020, a total of 756 SARS-CoV-2 infections were diagnosed at the S.Orsola Hospital in Bologna (Emilia-Romagna region, Italy). During these 2 months, 26 cases of SARS-CoV-2 infections were diagnosed in patients with HIV-1 infection. Therefore, the overall prevalence of coinfection of SARS-CoV-2 and HIV-1 among all cases of COVID-19 diagnosed in this period was 3.4% [interquartile range (IQR), 2.1–4.8]. The incidence of SARS-Cov-2 infection among HIV-positive persons referring to our hospital as of 30 April 2020 was 0.94% (IQR, 0.67–1.19).

Characteristics, treatments and outcomes of 26 HIV-infected patients with COVID-19 are summarized in Table 1. All patients gave informed consent. Overall, 19 (73%) were men, median age was 53.8 years (range, 28–80), all were currently treated with combination antiretroviral therapy (cART), and 22 subjects (85%) had a plasma HIV RNA below 50 copies/mL. Reported CD4 + T lymphocyte count and HIV RNA value were the last measurements before the COVID-19 diagnosis. Current CD4 + lymphocyte count was above 350 cells/mm^3^ in 22 individuals (85%), 5 (19%) had a previous diagnosis of an AIDS-defining condition, and current cART included one boosted protease inhibitor (PI) in 6 cases (23%). Nineteen patients (73%) had one or more comorbidities.

**Table 1:** Demographic data, clinical characteristics, treatment, and outcomes of patients with COVID-19 and HIV infection

Patients, *n*	26
Demographics, HIV baseline status and comorbidites	
Men, *n* (%)	19 (73.1)
Caucasian, *n* (%)	25 (96.1)
Age (years), median (IQR)	53.8 (42.5–64.7)
HIV transmission risk category, *n* (%)	
IDU	4 (15.4)
MSM	14 (53.8)
Heterosexual	8 (30.8)
Current CD4 + lymphocyte count (cells/mm^3^), median (IQR)	566 (304–821)
Patients with CD4 + lymphocyte count < 350 cells/mm^3^, *n* (%)	4 (15.4)
Patients with HIV RNA < 50 copies/mL, *n* (%)	22 (84.6)
Patients with AIDS diagnosis, *n* (%)	5 (19.2)
Duration of HIV infection (years), median (IQR)	16.2 (8.4–25.3)
Current ART	
NNRTIs, *n* (%)	13 (50)
Boosted PIs, *n* (%)	6 (23.1)
INSTIs, *n* (%)	11 (42.3)
Patients with one or more comorbidities, *n* (%)	19 (73.1)
Patients with arterial hypertension, *n* (%)	11 (42.3)
Patients with diabetes mellitus, *n* (%)	4 (15.4)
Patients with BMI > 30 kg/m^2^, *n* (%)	4 (15.4)
Patients with asthma or chronic respiratory diseases, *n* (%)	3 (11.6)
Patients with hypothyroidism, *n* (%)	3 (11.6)
Clinical presentation at diagnosis	
Diagnosis	
Upper respiratory tract infection, n. (%)	20 (76.9)
Interstitial pneumonia, n. (%)	6 (23.1)
Interstitial pneumonia with ARDS, n. (%)	0
Duration of symptoms before diagnosis (days), median (IQR)	4.2 (2.1–6.5)
Temperature > 38 °C, *n* (%)	19 (73.1)
Cough, *n* (%)	14 (53.8)
Myalgia, *n* (%)	11 (42.3)
Fatigue, *n* (%)	11 (42.3)
Dyspnoea, *n* (%)	3 (11.6)
Respiratory rate > 20 (breaths per min), *n* (%)	4 (15.4)
O_2_ saturation in ambient air < 95%, *n* (%)	7 (26.9)
PaO_2_/FiO_2_ ratio < 300, *n* (%)	2 (7.7)
White blood cell count (cells per 10^6^/L), median (IQR)	7.61 (3178–12,339)
Lymphocyte count (cells per 10^6^/L), median (IQR)	1555 (780–2331)
Patients with lymphocyte count < 1000 cells per 10^6^/L, *n* (%)	12 (46.1)
Patients with platelet count < 150,000 cells per 10^6^/L, *n* (%)	10 (38.5)
LDH (U/L), median (IQR)	618 (309–1108)
C-reactive protein (mg/dL), median (IQR)	4.2 (0.71–9.2)
Treatment and outcomes	
Hospitalization, *n* (%)	5 (19.2)
Admission to an ICU, *n* (%)	0
Non-invasive mechanical ventilation, *n* (%)	0
Darunavir/ritonavir or darunavir/cobicistat, *n* (%)	12 (46.1)
Hydroxychloroquine, *n* (%)	13 (50)
Azithromycin, *n* (%)	6 (23.1)
Enoxaparin, *n* (%)	6 (23.1)
Tocilizumab, *n* (%)	0
Corticosteroids, *n* (%)	0
Recovery, *n* (%)	22 (84.6)
Clinical improvement, *n* (%)	4 (15.4)
Death, *n* (%)	0
Duration of hospitalization (days), median (IQR)	9.2 (6.5–12.3)
Total duration of symptoms (days), median (IQR)	7.5 (4.9–10.2)

*IQR* interquartile range, *IDU* injection drug users, *MSM* men who have sex with men, *DRMs* drug resistance mutations, *NNRTIs* non-nucleoside reverse transcriptase inhibitors, *PIs* protease inhibitors, *INSTIs* integrase strand transfer inhibitors, *BMI* body mass index, *ARDS* acute respiratory distress syndrome, *LDH* lactic dehydrogenase, *ICU* intensive care unit

Diagnosis of COVID-19 was made by detection of SARS-CoV-2 RNA in oro- and/or naso-pharyngeal swab specimens by real-time RT-PCR targeting regions in the *N* gene, following the US CDC protocol. Clinical diagnosis was represented by upper respiratory tract infection in 20 cases (77%) and interstitial pneumonia in 6 (23%). At diagnosis, the median duration of symptoms was 4.2 days, and most frequent symptoms were fever > 38 °C, cough, fatigue, myalgia, and tachypnea. A reduction in O_2_ saturation < 95% in ambient air was present in 7 subjects (27%), but only 2 patients (8%) had an initial respiratory failure with a PaO_2_/FiO_2_ ratio < 300 at arterial blood gas analysis.

Only 5 patients (19%) with interstitial pneumonia were hospitalized, while other 21 subjects (81%) spent their disease period at home. The five hospitalized patients had a median age of 55.1 years (IQR, 46.2–68.3) and a median CD4 + lymphocyte count of 622 cells/mm^3^ (IQR, 458–814). All hospitalized patients had current CD4 + lymphocyte count above 350 cells/mm^3^. At diagnosis, 6 patients (23%) were receiving a PI-based cART, including darunavir–cobicistat in 5 cases and darunavir–ritonavir in one case. Sixteen patients (61.5%) were receiving a cART including tenofovir disoproxil fumarate or tenofovir alafenamide. A transitional change in cART was made in other 6 patients who were treated with a non-boosted PI-based regimen, because of the potential activity of HIV PIs against the Coronavirus protease. So, darunavir/cobicistat replaced rilpivirine in 5 cases and efavirenz in one case. With regard to other drug treatments, we prescribed hydroxychloroquine in 13 subjects (50%) and enoxaparin in 6 (23%), while tocilizumab and corticosteroids were not employed.

A clinical recovery was obtained in 22 patients (85%) and a clinical improvement in the remaining 4 (15%), while there were no admissions to ICU and no deaths. The five hospitalized patients were discharged after a median of 9.2 days, and the median global duration of symptoms before recovery in all observed patients was 7.5 days.

## Discussion

Clinical course and outcome of COVID-19 among patients with HIV-1 infection are still unknown. Some authors have speculated that HIV-1-infected patients on cART may have a lower risk for COVID-19 and related complications because of the in vitro activity of some antiretroviral drugs against SARS-CoV-2 and their defective cellular immunity, leading to a decreased possibility of cytokine dysregulation and consequent severe lung damage [12, 13]. On the contrary, other authors have suggested an increased risk for COVID-19 due to HIV-1-related immunosuppression [14].

In some recent cohort studies, HIV-infected persons hospitalized for COVID-19 had similar clinical characteristics and outcomes with other hospitalized cohorts of HIV-uninfected patients, and risk of COVID-19 and severe disease in suppressed HIV-positive people seems to be comparable with the general population [5–11].

In our experience, HIV-infected patients accounted for 3.4% of all patients with diagnosis of SARS-CoV-2 infection made at our hospital during the observation period. This prevalence is higher than that reported by Blanco JL et al. at the Hospital Clinic of Barcelona, Spain (0.92%) [7], but these authors described only COVID-19 patients co-infected with HIV-1 among consecutive subjects admitted to their hospital, and not co-infected patients who were not hospitalized. Moreover, not all HIV-positive patients of our clinics were tested for SARS-CoV-2, and probably there were many cases of asymptomatic SARS-CoV-2 infections among HIV-positive people, likewise in the general population, so these data should be considered with great caution.

In an observational prospective study performed at the Hospital Cajal of Madrid, Spain, Vizcarra P et al. [8] have described 51 HIV-infected patients with COVID-19 and compared their characteristics with 1,288 HIV-positive subjects without COVID-19. In this study, clinical course, radiological findings, and outcome of SARS-CoV-2 infection in HIV-positive persons were similar to those reported in the general population, and HIV-infected patients with COVID-19 had a higher prevalence of comorbidities (mostly hypertension and diabetes) than HIV-infected subjects without COVID-19. The incidence of COVID-19 among HIV-infected people reported by Vizcarra P et al. was 1.2%, or rather slightly higher in comparison with our cohort.

In a German series of 33 HIV-infected patients with COVID-19, 91% of subjects recovered and 76% had a mild form of disease, so morbidity and mortality of COVID-19 were comparable to those reported in other cohorts of HIV-negative patients [9].

In a case series of 31 HIV-infected patients hospitalized for COVID-19 in a large tertiary care centre in New York City [10], clinical features and outcomes were similar to those described in other hospitalized cohorts. The overall prevalence of COVID-19 among HIV-infected people (approximately 1.4%) was slightly lower than that reported in our cohort. In this study, all patients were on stable cART with undetectable HIV viral load, and most subjects had CD4 + T cell count above 200 cells/mm^3^, as much as observed in another case series of 27 HIV-positive persons with COVID-19 in New Jersey [11]. In another report of 88 HIV-infected patients with COVID-19 hospitalized in New York City, there were no differences in adverse outcomes in comparison with a demographically similar patient group. Moreover, HIV-positive patients had greater proportions of smoking and comorbidities than similar HIV-negative comparators [15]. Therefore, in these cohorts, SARS-CoV-2 infections do not seem to act as an opportunistic pathogen in the setting of HIV disease.

In our study, the median age of patients was 54 years and 23 subjects (88%) were younger than 70 years, but they had a significant prevalence of chronic comorbidities (73%), represented mostly by hypertension, diabetes and obesity, such as for HIV-1-negative COVID-19 patients [1–3]. Our patients showed in most cases a good immunological condition, so a low CD4 + lymphocyte count did not seem to be associated with an increased risk of COVID-19, while comorbidities were often present. A current PI-based cART was present in only 6 persons, and in other 6 cases, the antiretroviral regimen was adapted to a PI-based combination, even though clinical data about efficacy of HIV-1 PIs in patients with COVID-19 are still inconclusive.

In a few case reports, lopinavir/ritonavir associated with adjuvant drugs for interstitial pneumonia in COVID-19 patients had a more evident therapeutic effect than adjuvant drugs alone in improving clinical symptoms and reducing the SARS-CoV-2 viral load [16, 17]. However, in a randomized, controlled trial involving 199 Chinese patients with COVID-19 and treated with lopinavir/ritonavir for 14 days in addition to standard care or standard care alone, no significant benefit was observed in the lopinavir/ritonavir group. In detail, the PI-based treatment was not associated with a difference from standard care in the time to clinical improvement, mortality, and duration of detectable SARS-CoV-2 viral load [18]. No clinical data are currently available about other HIV PIs as treatment of COVID-19. A case series provided preliminary evidence that darunavir at the usual dosage did not prevent SARS-CoV-2 infection in HIV-1-positive persons [19].

In conclusion, from our experience, SARS-CoV-2 infection, which had among HIV-1-infected patients a clinical presentation comparable or milder than general population, was not more frequent or more serious among subjects with low CD4 + lymphocyte count, and was frequently associated with chronic comorbidities. Further, enlarged cohort studies are needed to better understand risk and clinical course of COVID-19 among HIV-infected people.
